# The TP53-Related Signature Predicts Immune Cell Infiltration, Therapeutic Response, and Prognosis in Patients With Esophageal Carcinoma

**DOI:** 10.3389/fgene.2021.607238

**Published:** 2021-06-16

**Authors:** Hongpan Zhang, Zheng Huang, Yangguang Song, Zhihao Yang, Qi Shi, Kaige Wang, Zhiyu Zhang, Zheng Liu, Xiaobin Cui, Feng Li

**Affiliations:** ^1^Key Laboratory for Xinjiang Endemic and Ethnic Diseases, Department of Pathology, Shihezi University School of Medicine, Shihezi, China; ^2^Department of Pathology and Medical Research Center, Beijing Chaoyang Hospital, Capital Medical University, Beijing, China

**Keywords:** oesophageal carcinoma, TP53, signature, immune cell infiltration, therapeutic responses

## Abstract

TP53 mutation (TP53^MUT^) is one of the most common gene mutations and frequently occurs in many cancers, especially esophageal carcinoma (ESCA), and it correlates with clinical prognostic outcomes. Nevertheless, the mechanisms by which TP53^MUT^ regulates the correlation between ESCA and prognosis have not been sufficiently studied. Here, in the current research, we constructed a TP53^MUT^-related signature to predict the prognosis of patients with esophageal cancer and successfully verified this model in patients in the TP53 mutant group, esophageal squamous cell carcinoma group, and adenocarcinoma group. The risk scores proved to be better independent prognostic factors than clinical features, and prognostic features were combined with other clinical features to establish a convincing nomogram to predict overall survival from 1 to 3 years. In addition, we further predicted the tumor immune cell infiltration, chemical drugs, and immunotherapy responses between the high-risk group and low risk group. Finally, the gene expression of the seven-gene signature (AP002478.1, BHLHA15, FFAR2, IGFBP1, KCTD8, PHYHD1, and SLC26A9) can provide personalized prognosis prediction and insights into new treatments.

## Introduction

Esophageal carcinoma (ESCA) is the seventh most common malignant tumor globally (Bray et al., 2020), with a 5-year survival rate ranging from 10 to 25% (Stein et al., 2005). This type of carcinoma consists of two main subtypes: esophageal adenocarcinoma (ESAD) and esophageal squamous cell carcinoma (ESCC). The incidence of both types of cancer is increasing year on year, so this study investigated the two together (Wei et al., 2015; Deng et al., 2018). Despite a variety of clinical treatments, the 5-year overall survival (OS) rate of this disease is still low (Suntharalingam et al., 2017; Huang and Yu, 2018). Recently, a handful of evidence has indicated that the number of immune-inflammatory cells in the tumor microenvironment (TME) may be associated with malignant phenotypes of ESCA (Lin et al., 2016; Kelly, 2017), thereby promoting the production and development of the population of ESCA (Zhao et al., 2020). Therefore, we need to further reveal the possible function and mechanism by which this TP53-related signature is mediated in ESCA.

P53 is a well-known suppressor gene that has multiple antitumor functions, including apoptosis, senescence, cell cycle arrest, DNA repair, and autophagy response (Hong et al., 2014; Zhang et al., 2017; Lundsten et al., 2020; Shi et al., 2020). Aside from the fundamental role of p53 in suppressing tumor initiation, abnormalities in TP53 (TP53 mutation status) are the most common genetic alterations observed, resulting in abnormal cell growth and other oncogene functions in human cancers, including non-small-cell lung cancer, breast cancer, liver cancer, and ESCA (Silwal-Pandit et al., 2017; Giacomelli et al., 2018; Jiao et al., 2018; Kang et al., 2018; Long et al., 2019; Xu et al., 2020). A wealth of published articles also show that TP53^Mut^ is significantly linked to patient outcomes by effectively modulating cancer cells to secrete cytokines and chemokines (Canale et al., 2020), supporting cancer cell onset and dissemination by establishing the TME (Ham et al., 2019; Pathak et al., 2015). For instance, TP53^Mut^ was reported to induce CXCL5, CXCL8, and CXCL12, which promoted cell migration and invasion *in vitro*, thus confirming that the secretion of pro-angiogenic factors and chemokines is a gain-of-function for TP53^Mut^ (Ignacio et al., 2018). Intriguingly, TP53^Mut^ literature associated with the tumor microenvironment (TME) has not been well reported in ESCA, and it is still unclear whether the regulation of immunity in ESCA depends on TP53 mutation. Therefore, therapeutic strategies targeting mutated TP53, which most patients with ESCA carry, may present a novel perspective for understanding the development of ESCA.

In this paper, we aimed to construct a TP53-related prognostic signature and to divide patients into two subtypes with different responses to chemotherapy. This was found to play a pivotal role in immune-related biological function in ESCA development. Overall, our studies highlight a new regulatory possibility for ESCA, which could contribute to the development of a therapeutic strategy for patients with esophageal carcinoma.

## Materials and Methods

### Patients and Specimens

The RNA-Seq data, somatic mutation data, and corresponding clinical data with ESCA patients were obtained from TCGA (February 1st, 2020^1^) (Tomczak et al., 2015). We collected 171 samples with gene expression data, including 160 tumor tissue samples, 11 normal samples, and 181 somatic mutation data, from the TCGA database. Somatic mutation data of 396 esophageal adenocarcinoma cases and 291 esophageal squamous cell carcinoma cases were obtained from the ICGC database^2^ (Banks and Lopezotín, 2010). Gene set enrichment analysis (GSEA) (Kuleshov et al., 2019; Reimand et al., 2019) was performed to investigate the potential mechanisms of TP53 mutation and wild-type TP53. The number of random sample permutations was set at 1,000, and the significance threshold was set at *p* < 0.05.

### Identification of Differentially Expressed Genes

Perl and R scripts were used for all data processing and normalized analysis and usage. Furthermore, the mRNA expression profile was analyzed to screen differentially expressed genes (DEGs) between the ESCA and normal samples. Dysregulated genes in the two groups were identified by using the edgeR package (Robinson et al., 2010). A | log 2fold change (log2FC) | > 2 and adjusted *P* < 0.05 were set as the threshold of differences. In addition, we divided the data into two groups based on the TP53 wild-type and mutated types; the mRNA expression profile was also analyzed to screen differentially expressed genes (DEGs) between these group. A | log2fold change (log2FC) | > 1 and adjusted *P* < 0.05 were set as the threshold of differences. By intersecting with the above DEGs, target genes were eventually achieved.

### Constructing the Prognosis Signature

The univariate Cox proportional hazard regression method was used to assess the relationship between DEGs and OS of patients. The 12 candidate genes (*P* < 0.05) were selected for stepwise multivariate Cox regression fitting. The remaining OS-related genes were adjusted by the stepwise multivariate Cox regression model. Furthermore, we identified those genes that fit in the model were independently associated with OS. Finally, according to the median risk score, patients were divided into a high-risk group and a low-risk group for comparison, and the log-rank test was used to compare the survival difference between the two groups.

### Independent Prognostic Role of the Multigene Signature and Construction of the Nomogram

To validate whether the prognostic signature could be independent among clinical parameters, including sex, age, tumor grade, TNM stage, and risk score, univariate and multivariate analyses were performed using the Cox regression model method with a forward stepwise procedure. Statistical significance was defined as a *P*-value < 0.05. All data were processed using the R package “survival ROC.” Additionally, a nomogram was established based on the independent prognostic factors identified by multivariate analysis to investigate the 1–, 2–, and 3-year OS of ESCA patients. The nomogram and calibration plots were generated using the RMS R package (Version: 5.1–3).

### Immunotherapeutic and Chemotherapeutic Response Prediction

The programmed cell death 1 (PCDC1, also known as PD-1)/CD274 molecule (CD274, also known as PD-L1) and cytotoxic T-lymphocyte associated protein 4 (CTLA-4) pathways have been implicated in tumor immune evasion, and therefore, immune checkpoint inhibitors targeting PD-1 and CTLA-4 can thereby enhance antitumor immunity. We used the Tumor Immune Dysfunction and Exclusion (TIDE) algorithm and subclass mapping to predict clinical responses to immune checkpoint inhibitors as previously described (Jiang et al., 2018; Fu et al., 2020). As chemotherapy is commonly used to treat ESCA, we used the R package “pRRophetic” to estimate the chemotherapeutic response determined by the half maximal inhibitory concentration (IC50) of each ESCA patient on the Genomics of Drug Sensitivity in Cancer (GDSC) website (Garnett et al., 2012; Yang et al., 2012; Orio et al., 2016).

### Estimation of Tumor-Infiltrating Immune Cells

CIBERSORT is an analytical tool developed by Newman et al. to provide an estimation of the abundances of member cell types in a mixed cell population using gene expression data, and it is highly consistent with ground-truth estimations in many cancers (Newman et al., 2015). Hence, we used a combination of the normalized esophageal cancer gene expression matrix and the LM22 signature matrix to estimate the scores of 22 human hematopoietic cell phenotypes between high- and low-risk patients. For each sample, the sum of all estimated immune cell type scores is equal to 1. The R package “Genefilter” was used to screen each sample, and the threshold was set at a *p*-value < 0.05. The final CIBERSORT output was subsequently analyzed.

### Statistical Analyses

Statistical analyses were performed with R software (Version 3.6.1). Univariate and multivariate Cox regression models were used to evaluate prognostic significance. The overall survival (OS) time of the risk groups was analyzed using Kaplan-Meier (KM) survival analysis, followed by a log-rank test. The sensitivity and specificity of the model were evaluated using ROC curves. Additionally, we verified the confidence of the model using test data sets and entire data sets. Recently, hazard ratios (HRs) and 95% confidence intervals (CIs) were used to describe the relative risk. A *P*-value < 0.05 was regarded as a statistically significant difference.

## Results

### TP53 Mutations in ESCA

**FIGURE 1:**
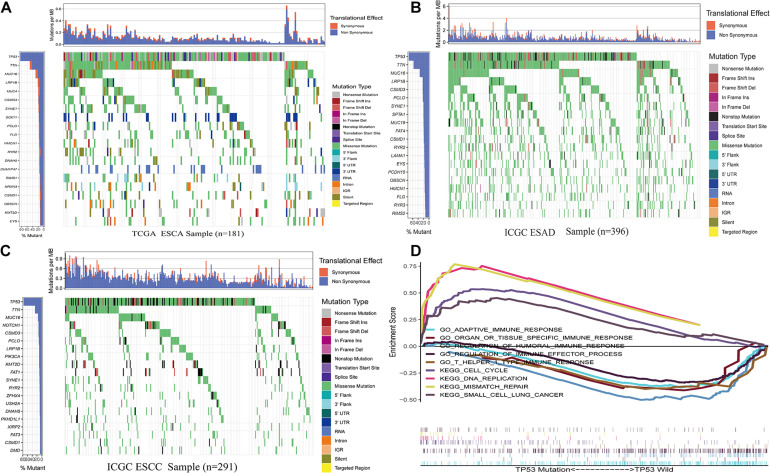
Somatic mutation analysis. **(A)** Overview of somatic mutations in all samples in the ESCA TCGA cohorts. **(B)** Mutations in ESAD samples in the ICGC cohorts. **(C)** Mutations in ESCC samples in the ICGC cohorts. **(D)** GSEA of samples with and without TP53 mutations. Abbreviations ESAD: esophageal adenocarcinoma, ESCC: esophageal squamous cell carcinoma.

**FIGURE 2:**
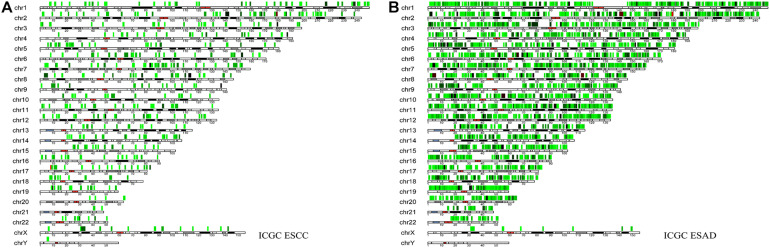
The location of various somatic mutations in esophageal squamous cell carcinoma **(A)** and esophageal adenocarcinoma on chromosomes **(B)**.

From the TCGA data, TP53 mutation in ESCA was found to be extraordinarily high (83%; Figure 1A). Previous studies have shown that TP53^MUT^ in ESCA plays a crucial role in patient prognosis and tumor-promoting phenotypes *in vivo* and *in vitro* (Ralhan et al., 2000; Makino et al., 2010; Wong et al., 2013). Similar to these observations, we also identified the TP53 mutation status of ESAC in the ICGC database, which occurred at a high frequency in the TCGA data (ranked first, Figures 1B,C). The location and frequency of the mutation in ESCA are shown in Figures 2A,B. Moreover, GSEA was performed to participate in gene ontological (GO) enrichment and KEGG pathway analysis to explore insights into TP53 mutation functionality. The results illustrated that samples with and without TP53 mutations were obviously clustered in KEGG_DNA_REPLICATION, KEGG_MISSMATCH_REPAIR; KEGG_CELL_CYCLE, KEGG_ SMALL_CELL_LUNG_CANCER, GO_ADAPTIVE_IMMUNE _RESPONSE, GO_ORGAN_OR_TISSUE_SPECIFIC_IMMUNE _RESPONSE (Wu et al., 2020), GO_REGULATION _OF_HUMORAL_IMMUNE_RESPONSE, GO_T_HELPER_1_ TYPE_IMMUNE_RESPONSE, and GO_REGULATION_OF_ IMMUNE_EFFECTOR_PROCESS, suggesting that the gene associated with TP53^MUT^ can have an immunomodulatory effect on ESCA (Figure 1D).

### Identification of DEGs-3 in ESCA Patients

**FIGURE 3:**
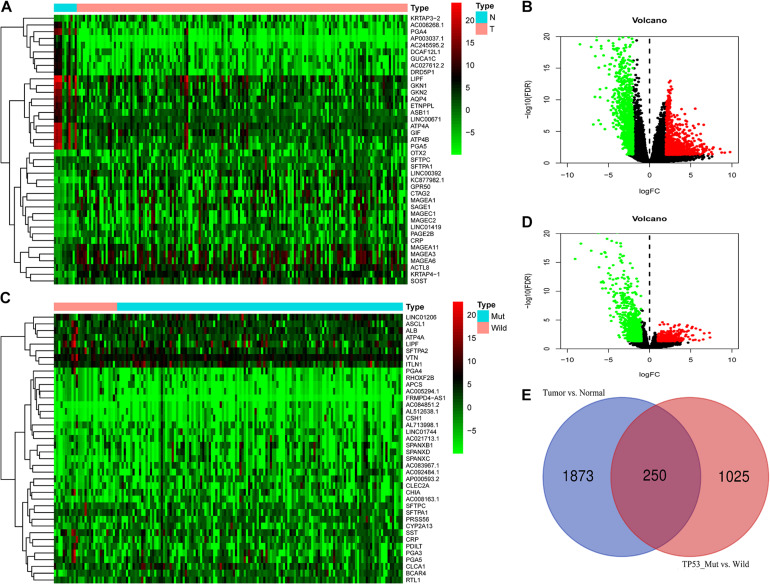
Differential expression analysis **(A,B)** DEG-2 between ESCA and adjacent tissues. **(C,D)** DEGs-1 between the ESCA TP53^WT^ and TP53^MUT^ cohorts. **(E)** DEGs-3: intersections of DEGs-1 and DEGs-2. Abbreviations ESCA: esophageal carcinoma.

A total of 2123 DEGs-2 were incorporated into our subsequent analysis (log2| fold change| > 2 and adj. *P* < 0.05) between the ESCA and adjacent tissues (Figures 3A,B). Furthermore, we classified ESCA patients with TP53^WT^ and TP53^MUT^ cohorts and identified 1275 DEGs-1 above them ((log2| fold change| > 1 and adj. *P* < 0.05) (Figures 3C,D). Ultimately, by performing the intersections of DEG-1 and DEG-2 as DEG-3, 250 common DEGs were chosen (Figure 3E).

### A Prognostic Signature Based on the DEGs-3

**FIGURE 4:**
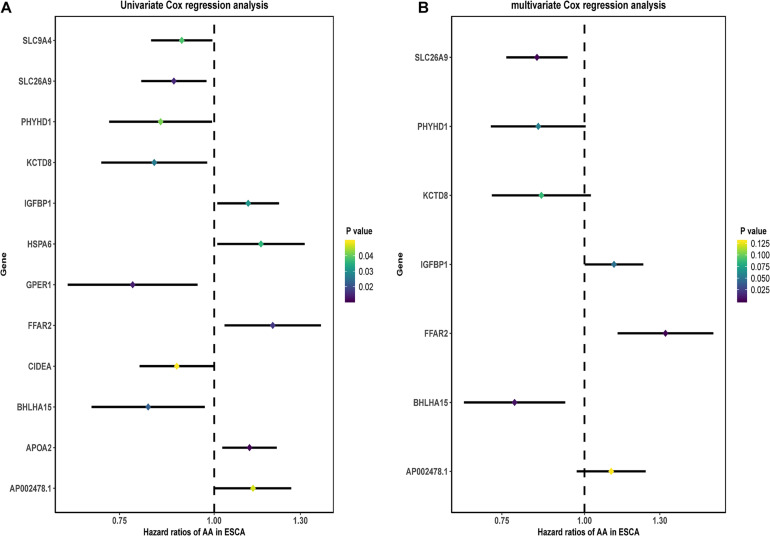
Construction of the DEG-based prognostic signature. **(A)** Prognostic value of DEG-3 according to univariate Cox regression analysis. **(B)** Prognostic value of DEG-3 according to multivariate Cox regression analysis.

**FIGURE 5:**
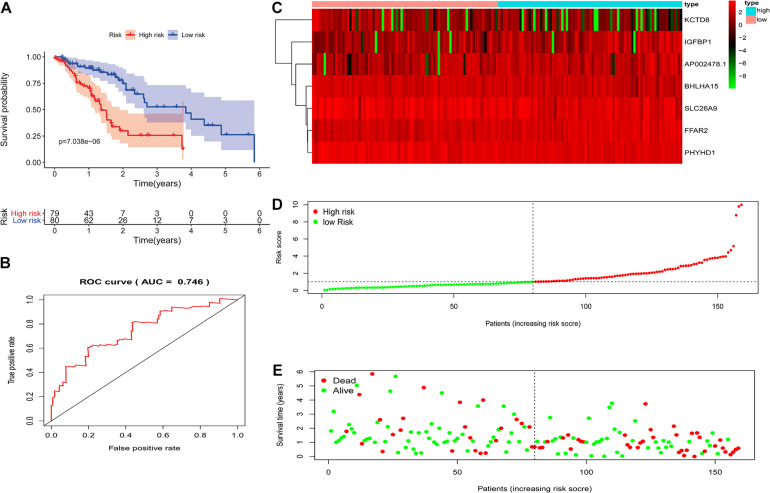
The prognostic values of the signature. **(A)** Kaplan-Meier survival curves of the relative overall survival of high- and low-risk patients. **(B)** ROC curve analysis of the prognostic signature. **(C)** Heatmap of three-gene expression profiles in the high- and low-risk groups. **(D)** The distribution of the three gene-based risk scores. **(E)** Vital statuses of patients in the high- and low-risk groups.

**FIGURE 6:**
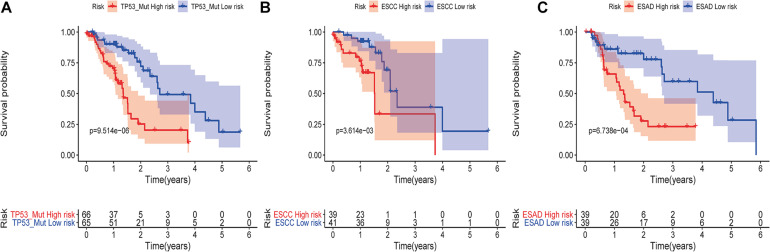
Kaplan-Meier analysis of overall survival in ESCA patients according to the risk model. Kaplan-Meier analysis of the TP53 mutation subgroup **(A)**; the ESCC subgroup **(B)**; and the ESAD subgroup **(C)**.

Based on the above DEGs-3, we screened these 250 candidate genes to evaluate the genetic prognostic markers using the univariate analysis. Among the 12 candidate variables (Figure 4A), the seven-gene prognosis prediction model (Table 1 and Figure 4B) was eventually filtered and was independently associated with prognosis. The risk score was calculated using a multivariate Cox proportional hazard regression model: risk score = 0.093^∗^ (AP002478.1 expression level) –0.243^∗^ (BHLHA15 expression level) + 0.282^∗^ (FFAR2 expression level) + 0.103^∗^ (IGFBP1 expression level) –0.150^∗^ (KCTD8 expression level)–0.160^∗^ (PHYHD1 expression level) –0.165^∗^ (SLC26A9 expression level). According to the model, ESCA patients were divided into high- and low-risk groups using the median risk score. Patients in the high-risk group had an obviously worse OS than their corresponding counterparts (*P* < 0.00001, log-rank test, Figure 5A). The longest survival time for the high-risk group was 4 years; in contrast, that for the low-risk group was 6 years. The time-dependent ROC curve results revealed that this seven-gene signature could strongly predict the OS of ESCA patients (AUC = 0.746; Figure 5B). The distribution of risk scores and vital statuses of patients sorted by survival time and the seven-genes expression heatmap were also consistent with these findings (Figures 5C–E). Moreover, in the subgroup analysis the gene signature showed better predictive power for patients in the TP53 mutant group, ESCC group, and ESAD group (Figures 6A–C), indicating that this model has good predictive ability in esophageal cancer.

**TABLE 1:** Model information.

**Id**	**Coef**	**HR**	**HR.95L**	**HR.95H**	***p-*value**
AP002478.1	0.092954	1.097411	0.973312	1.237333	0.128973
BHLHA15	–0.24329	0.784041	0.657294	0.935229	0.006845
FFAR2	0.281921	1.325673	1.122192	1.566051	0.000913
IGFBP1	0.102793	1.108262	1.000941	1.22709	0.047922
KCTD8	–0.15004	0.86067	0.724632	1.022246	0.087393
PHYHD1	–0.16087	0.851403	0.721925	1.004102	0.055963
SLC26A9	–0.1653	0.847641	0.76189	0.943044	0.002385

Among these seven genes, the coefficients of BHLHA15, KCTD8, PHYHD1, and SLC26A9 were negative, suggesting that they may have a survival promotion function, while the remaining genes seemed to be risk factors and were more highly expressed in the high-level group than in the low-level group.

### Independent Prognostic Role and Construction of a Nomogram Based on the Signature

**FIGURE 7:**
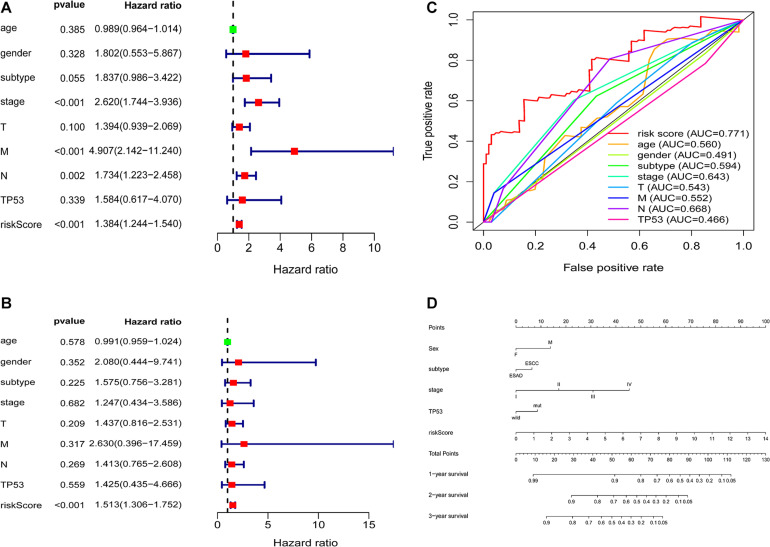
Correlation between the seven-gene signature and patient clinical characteristics **(A,B)** Univariate and multivariate Cox regression analyses of the correlations between the TP53-associated signature and clinical characteristics with overall survival. **(C)** ROC curve analyses of age, sex, subtype, stage, T stage, M stage, N stage, TP53, and prognostic risk model. **(D)** Nomogram for predicting the 1-, 3-, and 5-year overall survival of patients with ESCA.

Clinical information of ESCA patients, including sex, age, tumor subtype, TNM stage, TP53, and risk score, were included for further analysis. The univariate and multivariate analyses indicated that the risk score from the seven-gene signature was a specific predictor of OS (hazard ratio = 1.513, *P* < 0.001) (Figures 7A,B). The AUCs of the prognostic model, age, sex, subtype, stage, T stage, M stage, N stage, and TP53 were 0.771, 0.560, 0.491, 0.594, 0.643, 0.543, 0.552, 0.668, and 0.466, respectively (Figure 7C). Based on the above findings, we constructed a nomogram combined with our signature and clinical features (sex, subtype, stage, TP53, and risk score) to directly predict the 1-, 2-, and 3-year survival rates (Figure 7D).

### Therapeutic Responses for ESCA

**FIGURE 8:**
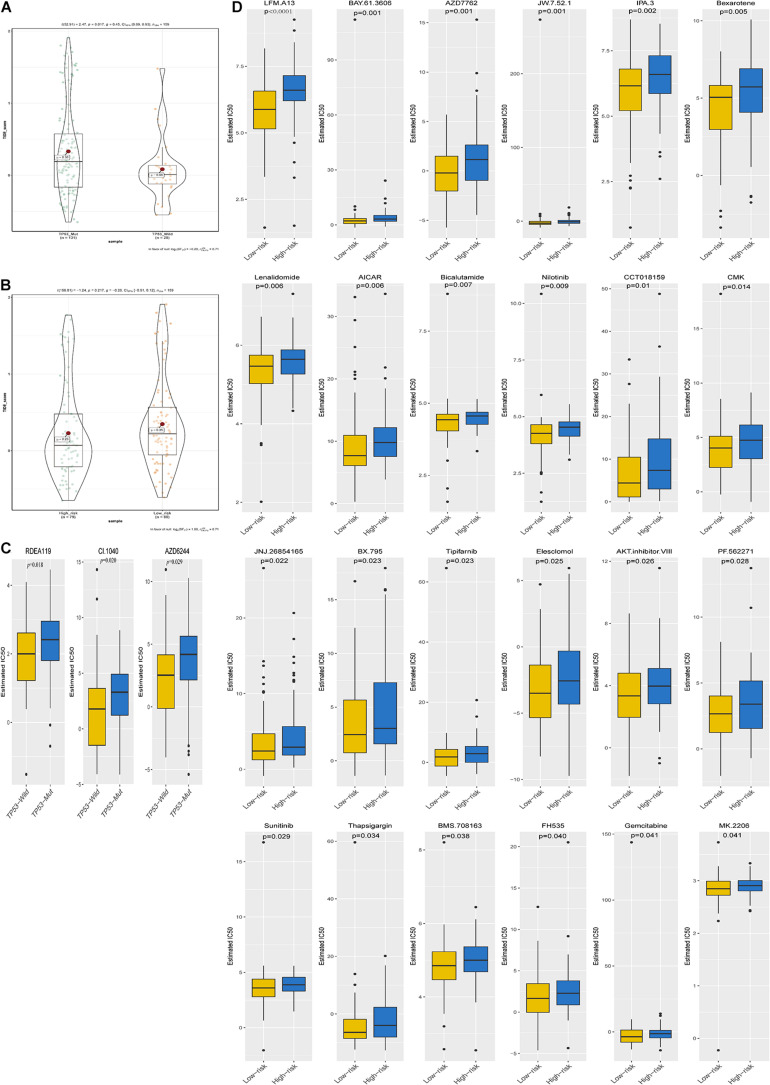
Immunotherapeutic and chemotherapeutic responses in the TP53^WT^ and TP53^MUT^ cohorts and the high- and low-risk patients with ESCA **(A,B)**. Immunotherapeutic response to anti-CTLA-4 and anti-PD-1 treatments in the high- and low-risk patients and the TP53^WT^ and TP53^MUT^ cohorts. **(C,D)** Differential chemotherapeutic responses in the TP53^WT^ and TP53^MUT^ cohorts and the high- and low-risk patients.

The TIDE score was significantly different between the TP53 mutant group and the wild-type group. Patients in the TP53 mutant group had higher TIDE scores (Figure 8A), but there was no significant difference in the scores between the high- and low-risk groups (Figure 8B). Subsequently, we explored changes in chemotherapy resistance and found that three chemotherapy and targeted drugs showed great differences in the estimated IC50 between the TP53^MUT^ and wild groups, and 24 chemotherapy and targeted drugs showed great differences in the estimated IC50 between the high-risk and low-risk groups, with patients in the mutant group or high-risk group having higher IC50 values (Figures 8C,D). Together, these results can provide better guidance for treatment selection of drug for *ESCA patients*.

### Immune Cell Infiltration Landscape in ESCA Patients

**FIGURE 9:**
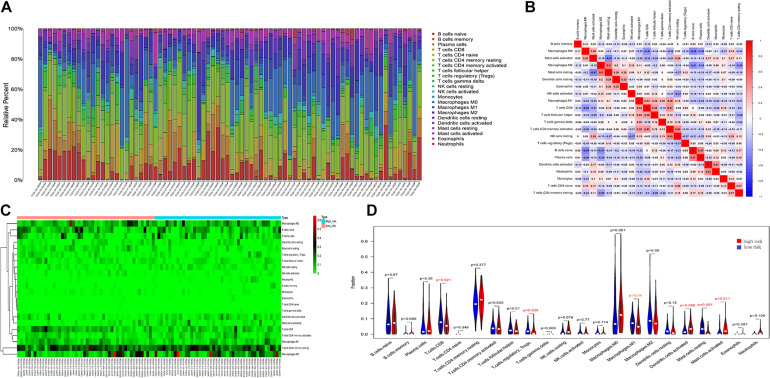
Immune cell infiltration landscapes in high- and low-risk patients with ESCA. **(A)** Relative level of immune cell infiltration in high- and low-risk patients. **(B)** Correlation matrix and **(C)** heatmap of the 22 immune cell proportions. **(D)** Differences in immune cell infiltration between high- and low-risk patients.

We investigated the difference between high-risk and low-risk patients via the CIBERSORT algorithm in 22 immune cell infiltrates of ESCA. The immune cells varied distinctly between samples (Figure 9A); moreover, immune cell proportions were weakly to moderately correlated (Figure 9B). The heat map also exhibited the difference in immune cell infiltration between the two groups, where the colors ranging from green to red represent the infiltration density from low to high (Figure 9C). The Wilcoxon rank-sum test was also accurately applied to explore this difference and found that several immune cells conferred a significantly low infiltration density in the high-risk groups, including T cells CD8 (*P* = 0.021), regulatory T cells (*P* = 0.030), M1 macrophages (*P* = 0.04), and resting mast cells (*P* = 0.001) (Figure 9D). In light of the above analysis, we hypothesized that this signature was linked to immune cell infiltrates and has implications for poor survival outcomes.

### Correlation Between Immune Cell Infiltration and Expression of Seven Genes

**FIGURE 10:**
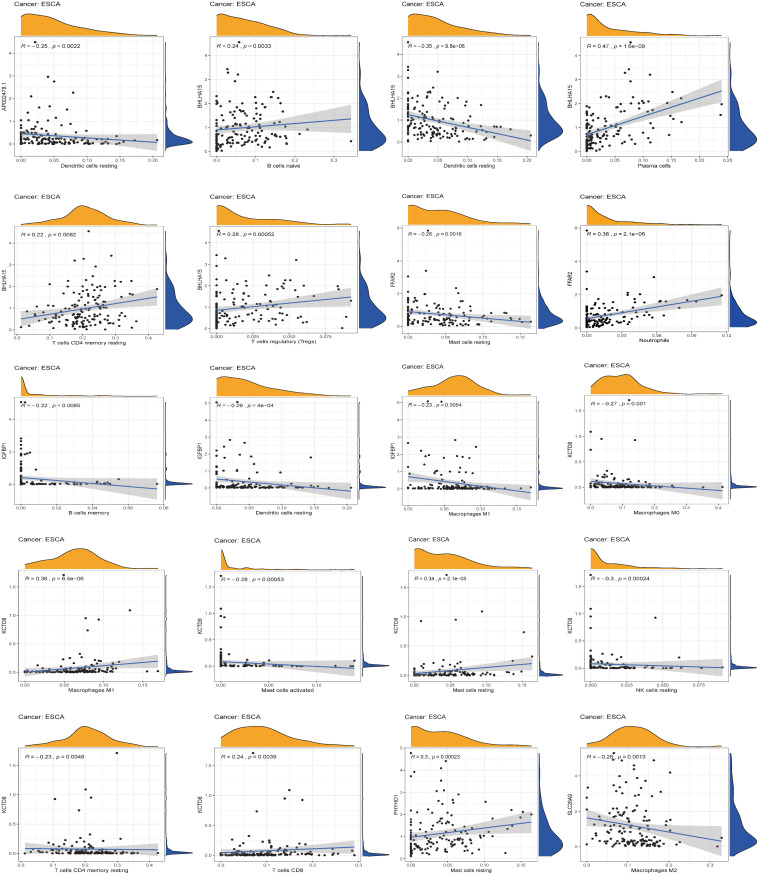
The relationship between the seven genes and immune cell infiltration.

After identifying the immunotherapeutic value of the signature, a significant correlation was found between the expression level of the seven genes and the level of immune infiltration for ESCA (Figure 10). Scatter plots were generated with partial Spearman’s correlation and analyzed for statistical significance. AP002478.1, BHLHA15, and IGFBP1 expression was significantly associated with resting dendritic cells (correlation = –0.25, –0.35, and –0.29, *p*-value < 0.01), while the expression of these genes was also significantly related to NK cell activation, M0 macrophages, M2 macrophages, follicular helper T cells, and plasma cell immune infiltration (*p*-value < 0.01).

## Discussion

ESCA remains one of the most malignant gastrointestinal cancers and is ranked sixth in cancer-related mortality worldwide (Domper Arnal et al., 2015). Despite advancements in diagnosis and treatment for ESCA in recent decades, its incidence and mortality have only been slightly reduced (Gupta and Kumar, 2017). TP53^MUT^ is involved in immunity by modulating apoptosis, antiviral defense, and the induction of type I IFN, thereby promoting cytokine production and immune checkpoint evasion in some cancer cells (Yao and Sherif, 2016; Moore et al., 2018; Li et al., 2020). However, the mechanisms by which TP53^MUT^ regulates the correlation between ESCA prognosis and TME have not been well reported. Therefore, it is essential to elucidate the TP53^MUT^-associated immune effect on ESCA.

In our current work, two groups of DEGs (DEGs-1 and DEGs-2) were obtained from ESCA tissues and matched normal specimens, as well as TP53^WT^ and TP53^MUT^ cohorts. We further intersected the two above groups to identify the TP53 mutations associated with ESCA. Strikingly, the GSEA results illustrated that mutated TP53 was obviously enriched in immune biological functions, suggesting that TP53^MUT^ can have an immunomodulatory effect on ESCA. Furthermore, we successfully constructed a signature to predict ESCA patient survival based on TP53^MUT^-linked genes, and the overall survival analysis showed a significant difference between the two groups, which had satisfactory specificity and sensitivity in the prediction of ESCA based on the various methods available. Aside from the above analyses, CIBERSORT can be used to evaluate the interaction between the 22 immune cell types and this signature that is closely related to the immune processes associated with human tumors. The high- and low-risk groups had obvious differences in terms of the types of immune infiltrating cells, such as CD8^+^ T cells, Tregs, M1 macrophages, resting mast cells, and resting dendritic cells. Taken together, the results indicate that this immune-related signature may be one of the factors leading to differences in patient prognoses.

It has been reported that TP53^MUT^-mediated biological processes are significantly associated with immune activities in multiple cancers. Researchers have found that adoptive transfer of wild-type TP53-specific T cell clones restricts the growth of mutant TP53-transformed MEFs *in vivo* (Hoffmann et al., 2002; Zwaveling et al., 2002). Behring et al. (2019) also determined that TP53 mutation in breast tumors is associated with immune inflammation-related gain of function and changes in tumor-associated macrophages. More importantly, we identified a number of TP53-mediated pathways whose activities were significantly associated with immune activities in ESCA. In addition, in the TP53^Mut^ group, the TIDE score was high, so they may be candidates for immune therapy (Lim et al., 2018). Unfortunately, we found that TIDE had no more substantial value in patients with high-risk ESCA than in their low-risk counterparts. This is mainly because there are fewer tumor species than in other tumors. Moreover, chemotherapy has been successful at treating ESCA (van den Ende et al., 2018), and patients of TP53-mut are more sensitive to the drugs RDEA119, CI.1040, and AZD6244 than their TP53-wild counterparts. Low-risk patients are more sensitive to 24 chemotherapy drugs and targeted drugs than their high-risk counterparts. Taken together, this work can serve as a reference for clinical decisions regarding suitable drugs, as patients in different groups are likely to show different immunotherapeutic responses.

The above results show that the poor prognosis in ESCA patients is mainly because of the reduction in immune activity and inhibited immune response in the TME, which can be targeted to overcome tumor immune suppression and enhance antitumor immunity. TP53^WT^ and low-risk patients with ESCA may benefit from immunotherapy and chemotherapy, while TP53^MUT^ and high-risk patients need different treatments. Nevertheless, some limitations of our investigation should be noted. First, the biological function of the seven identified genes, especially their association with immune *infiltration*, must be evaluated in functional assays. Second, only a limited amount of data was used for the performance assessment, so it is necessary to collect more robust datasets and to validate this signature in the future.

Furthermore, AP002478.1, a non-coding RNA identified in this study, lacks relevant research reports. BHLHA15 (also known as Mist1), a basic helix-loop-helix transcription factor, can give rise to cancers (Hayakawa et al., 2015; Sakitani et al., 2017). FFAR2 (also named GPR43) is a member of the G-protein-coupled receptor family that is expressed in leukocytes and the colon (Tan et al., 2014). The microbial metabolite-sensing receptor FFAR2 is critical for the regulation of immune cell function and the maintenance of gut homeostasis, and the loss of FFAR2 increases tumor burden in multiple models of tumorigenesis (Sivaprakasam et al., 2016; Pan et al., 2017; Kim et al., 2018). Insulin-like growth factor binding proteins (IGFBPs) are a family of secreted proteins that were originally characterized as passive carriers of insulin-like growth factors (IGFs) in the circulation with high affinity. The intranuclear roles of IGFBPs are transcriptional regulation, induction of apoptosis, and DNA damage repair, which point to their intimate involvement in tumor development, progression, and resistance to treatment (Baxter, 2014). Studies have shown that miR-454-3p overexpression can inhibit the expression of insulin-like growth factor 2 mRNA-binding protein 1 (IGF2BP1) at the protein and mRNA expression levels and thus inhibit the occurrence and development of esophageal cancer (Yan et al., 2020). Consistent with our study, IGF2BP1 is a poor prognostic factor for esophageal cancer (Yan et al., 2020). There have been few studies on KCTD12 in cancer. Some researchers have reported that hypermethylation of KCTD8 is associated with the occurrence and development of breast cancer (Faryna et al., 2012), which is consistent with our study. PHYHD1, phytanoyl-CoA dioxygenase domain containing 1, is a putative ortholog of Xenopus phytanoyl-CoA dioxygenase-like (Furusawa et al., 2016). Reports of PHYHD1 in cancer are rare, and bioinformatics analyses have shown that PHYHD1 is associated with the prognosis of laryngeal squamous cell carcinoma (Cui et al., 2020). SLC26 (solute carrier family 26) proteins function as anion transporters and/or channels (Alper and Sharma, 2013). Nevertheless, information regarding the role of SLC26A9 in cancer is lacking.

## Conclusion

In conclusion, for the first time, we established a gene signature based on TP53 mutation status. This signature was found to be an independent prognostic factor for ESCA patients and showed promising sensitivity and specificity for survival predictions. In addition, immune cell infiltration differences were observed in the ESCA immune microenvironment, which could guide drug treatment strategies.

## Data Availability Statement

Publicly available datasets were analyzed in this study. This data can be found here: https://portal.gdc.cancer.gov/repository.

## Author Contributions

HZ, ZH, and YS designed the experiments, and wrote and revised the paper. ZY and QS designed the experiments and analyzed the data. KW, ZZ, and ZL collected the data and modified the language of the article. XC and FL conducted the experiments. All authors contributed to the article and approved the submitted version.

## Conflict of Interest

The authors declare that the research was conducted in the absence of any commercial or financial relationships that could be construed as a potential conflict of interest.

## Footnotes

1 https://cancergenome.nih.gov/

2 https://icgc.org/
